# PD-1^+^ melanocortin receptor dependent-Treg cells prevent autoimmune disease

**DOI:** 10.1038/s41598-019-53297-w

**Published:** 2019-11-15

**Authors:** Fauziyya Muhammad, Dawei Wang, Alyssa Montieth, Stacey Lee, Janine Preble, C. Stephen Foster, Theresa A. Larson, Kai Ding, Justin D. Dvorak, Darren J. Lee

**Affiliations:** 1 Department of Microbiology and Immunology, University of Oklahoma Health Sciences Center, Oklahoma City, Oklahoma USA; 2 Department of Ophthalmology/Dean McGee Eye Institute, University of Oklahoma Health Sciences Center, Oklahoma City, Oklahoma USA; 3 Massachusetts Eye Research and Surgery Institute, Waltham, Massachusetts USA; 4 Ocular Immunology and Uveitis Foundation, Waltham, Massachusetts USA; 5 Harvard Medical School, Boston, Massachusetts USA; 6 College of Public Health, University of Oklahoma Health Sciences Center, Oklahoma City, OK USA

**Keywords:** Neuroimmunology, Translational research

## Abstract

Experimental autoimmune uveoretinitis (EAU) is a mouse model of human autoimmune uveitis marked by ocular autoantigen-specific regulatory immunity in the spleen. The melanocortin 5 receptor (MC5r) and adenosine 2 A receptor (A2Ar) are required for induction of post-EAU regulatory T cells (Tregs) which provide resistance to EAU. We show that blocking the PD-1/PD-L1 pathway prevented suppression of EAU by post-EAU Tregs. A2Ar induction of PD-1^+^FoxP3^+^ Tregs in uveitis patients was similar compared to healthy controls, but was significantly reduced with melanocortin stimulation. Further, lower body mass index correlated with responsiveness to stimulation of this pathway. These observations indicate an importance of the PD-1/PD-L1 pathway to provide resistance to relapsing uveitis and shows a reduced capacity of uveitis patients to induce Tregs when stimulated through melanocortin receptors, but that it is possible to bypass this part of the pathway through direct stimulation of A2Ar.

## Introduction

The third leading cause of blindness in Western countries is uveitis, with an incidence between 25.6 – 122 cases per 100,000 a year, and a prevalence of 69–623 cases per 100,000^1–3^. The most common anatomic location of uveitis is anterior uveitis, with 33% of these patients becoming chronic^4^. Approximately 17.6% of active uveitis patients experience transient or permanent vision loss, and it is estimated that glaucoma will occur in 12.5% of these patients^5^. As with other autoimmune diseases, autoimmune uveitis patients experience relapsing and remitting inflammation^6–8^. How to achieve prolonged remission is unknown, but a better understanding of the mechanisms contributing to the relapsing and remitting intraocular inflammation has the potential to develop novel more effective treatments for autoimmune uveitis.

Experimental autoimmune uveitis (EAU) is a mouse model of human autoimmune uveitis that resolves without further relapse^9–11^. In C57BL/6 J mice, resolution of EAU occurs at 75–90 day following immunization for EAU and at this point (post-EAU) regulatory immunity is found in the spleen^12–15^. This post-EAU regulatory immunity provides resistance to relapse, and can be transferred to mice to suppress uveitis when recipient mice are immunized for EAU^13–16^.

Programmed death receptor 1 (PD-1) was initially identified as a receptor on T cell hybridomas undergoing apoptosis^17,18^. Subsequent findings revealed that PD-1 is also expressed on T cells following stimulation, and on regulatory T cells (Tregs)^18^. The ligand for PD-1 is PD-L1 and PD-L2 that can be expressed by many leukocytes including T cells and antigen presenting cells (APC)^15,18,19^, and on ocular tissues such as corneal epithelial cells and retina pigmented epithelial cells^20,21^. Stimulation of PD-1 by its ligand inhibits TCR signaling through blocking the PI3K pathway, resulting in suppression of effector T cell function and the induction of Treg cell activity^18^.

The post-EAU regulatory immunity that provides resistance to EAU requires expression of the adenosine 2 A receptor (A2Ar) on T cells, and suppressor APC that express the melancortin 5 receptor (MC5r)^12,14,15^. When stimulated through MC5r, the suppressor APC up-regulate the ectoenzymes, CD39 and CD73, that hydrolyze ATP into adenosine^14^. The post-EAU suppressor APC activates Treg cells that express PD-1 and PD-L1 and suppress effector cytokines in a PD-1/PD-L1 dependent manner^15^. However, it has not been demonstrated if these post-EAU Tregs require the PD-1/PD-L1 pathway to functionally suppress EAU. Furthermore, while it has been demonstrated that uveitis patients express MC5r and A2Ar on monocytes and T cells at similar levels as healthy controls^12^, it has not been demonstrated that stimulation of this pathway can promote Treg cells.

In this report, we demonstrate that post-EAU PD-1^+^ CD25^+^CD4^+^ Treg cells from mice require the PD-1/PD-L1 pathway for functional suppression of EAU. Importantly, we show that uveitis patients express CD39 and CD73 at similar levels as healthy controls, but do not upregulate to the same extent with melanocortin and A2Ar stimulation in the classical monocyte subset. Further, the number of PD-1^+^FoxP3^+^CD25^+^CD4^+^ Tregs in uveitis and healthy groups were similar when stimulated through A2Ar, this suggests there is no defect in response to A2Ar stimulation in the uveitis cohort, but a significantly lower number of Tregs with melanocortin stimulation suggests the deficiency is in response to melanocortin stimulation. Taken together, these observations demonstrate an importance of the PD-1/PD-L1 pathway for the function of Tregs that provide resistance to relapse, and a defect in response to melanocortin stimulation correlates with autoimmune uveitis.

## Results

### The PD-1/PD-L1 pathway is necessary for ocular autoantigen-specific Treg suppression of EAU

**Figure 1:**
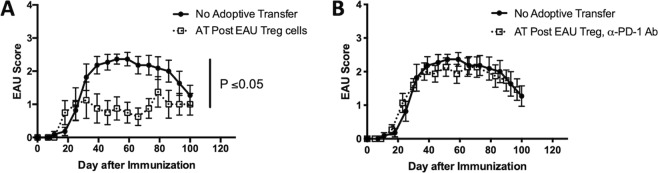
Effect of PD-1 blockade on functional suppression of post-EAU Treg cells. Spleen cells from post-EAU mice were collected and reactivated *in vitro* with IRBP and adoptively transferred to recipient mice immunized for EAU. The solid line with closed circles are EAU control mice that did not receive an adoptive transfer of spleen cells, the dashed line with open squares indicates mice were immunized for EAU and received spleen cells. One experimental group of mice received post-EAU spleen cells alone (**A**, n = 8), and another experimental group of mice received post-EAU spleen cells that were reactivated with α-PD-1 blocking antibody (**B**, n = 15). Each experiment consisted of 4–5 mice, and each experiment was repeated 2–3 times. Significance was determined by two-way ANOVA with Bonferroni post-test.

Since it has been previously demonstrated *in vitro* that ocular autoantigen-specific Treg cells suppress interphotoreceptor retinoid binding protein (residues 1–20) (IRBP)-specific inflammatory T cells in a PD-1/PD-L1 dependent manner^15^, we asked if these same Treg cells require the PD-1/PD-L1 pathway for functional suppression of EAU *in vivo*. When spleen cells from EAU-recovered mice were reactivated and transferred to recipient mice immunized for EAU, the recipient mice showed significantly suppressed EAU scores (Fig. 1A) as previously demonstrated^12–15^. In contrast, when PD-1 blocking antibody was added to the spleen cells at the reactivation step, the recipient mice showed no significant difference compared to EAU mice that did not receive a transfer of cells (Fig. 1B). These observations demonstrate that ocular antigen-specific Treg cells require the PD-1/PD-L1 pathway for functional suppression of EAU.

### Ocular autoantigen-specific Tregs do not induce suppressor APC

**Figure 2:**
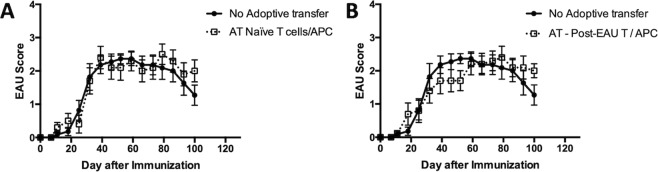
Effect of post-EAU Treg cells on the induction of suppressor APC. Spleen cells from post-EAU mice were collected and reactivated *in vitro* with IRBP, sorted, and incubated with APC from the spleen of unimmunized mice. Primed IRBP-specific T cells were collected from the popliteal lymph nodes and added to the APC from above, and transferred to recipient mice immunized for EAU. The solid line with closed circles represents EAU control mice that did not receive an adoptive transfer (AT) of cells. The dashed line with open squares indicates mice immunized for EAU that received cells. One group of mice received APC pre-incubated with T cells from unimmunized mice (**A**, n = 10), another group of mice received cells where APC were pre-incubated with sorted post-EAU Tregs (**B**, n = 10). Each experiment consisted of 5 mice, and each experiment was repeated 2 times. Significance was assessed by two-way ANOVA with Bonferroni post-test.

It has been reported that PD-1 stimulation on APC can promote suppressor function^18^ and Tregs from the spleen of post-EAU mice express both PD-1 and PD-L1^15^. Therefore, it is possible that ocular autoantigen-specific Tregs induce suppressor APC through the PD-1/PD-L1 pathway. We asked if ocular autoantigen specific Tregs promote suppressor APC by culturing the APC and PD-1^+^CD25^+^CD4^+^ Tregs together before the APC was used to activate ocular autoantigen-specific effector T cells. The APC and T cells were then transferred to recipient mice immunized for EAU. As expected, no suppression of EAU was observed in mice that received APC and ocular autoantigen-specific T cells (Fig. 2A). However, the APC that was pre-cultured with Tregs and used to activate ocular antigen-specific T cells also showed no suppression of EAU (Fig. 2B). These observations show that ocular autoantigen-specific Treg cells are unable to induce suppressor APC activity.

### PBMCs from uveitis patients exhibit an inflammatory cytokine profile

**Figure 3:**
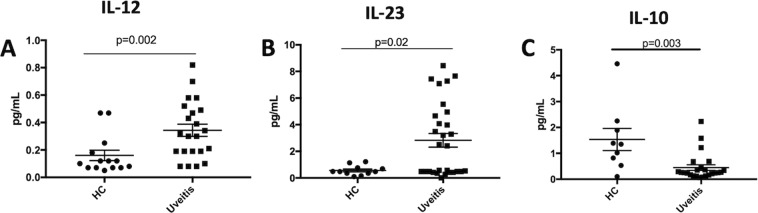
Cytokine profile in PBMCs from uveitis patients. PBMCs from uveitis patients or healthy donors were cultured for 48 hours in serum free media. After the 48-hour culture the supernatants were collected and cytokines were measured by Bioplex. The cytokines that were significantly different between PBMCs from health controls (HC) and uveitis patients (uveitis) is shown. The proinflammatory cytokines IL-12 (**A**) for HC (n = 14) and uveitis (n = 22) and IL-23 (**B**) HC (n = 11) and uveitis (n = 29), and regulatory cytokine IL-10 (**C**) are shown HC (n = 9) and uveitis (n = 24). Significance was assessed by nonparametric Mann-Whitney U test. Statistical significance (p < 0.05) is designated by *.

Our mouse findings demonstrate that mice with regulatory immunity capable of suppressing uveitis have a regulatory cytokine profile in the spleen^12–15^. In order to determine if there are similarities in the cytokine profile of uveitis patients with our mouse findings we compared the cytokine profile of uveitis patients with healthy volunteers. Blood was collected from uveitis patients or healthy controls. The inclusion criteria for uveitis patients was individuals with non-infectious uveitis. The inclusion criteria for healthy controls was individuals with no history of autoimmune disease or autoimmune uveitis. There was no significant difference between ages of healthy controls (HC) and uveitis (Table 1). Comparison of the type of uveitis included more anterior uveitis samples with posterior uveitis samples as the second most abundant, and the gender and age for each group is shown (Table 1). We also compared the treatment that each of the uveitis patients were taking. Immunomodulatory therapy (IMT) consisting of methotrexate, cellcept, and/or cyclosporine had the greatest number of patients, with patients taking steroids, IMT, biologics (rituximab, adalimumab, or infliximab), and NSAIDs (topical, bromfenac) as the second most abundant category (Table 2). We did not further stratify the treatment with the type of uveitis because there were no significant differences between each of the treatment groups (data not shown). We also compared active uveitis patients with suppressed uveitis patients that were defined as no inflammation in the previous two years from the time of sample collection, and found no significant differences between the two groups (data not shown). The group of patients that was off all medications was only n = 4, but the age was greater by at least a decade compared to other groups, and the patients on IMT or Biologics alone was skewed towards male. We collected PBMCs from a total of 22 HC and 45 uveitis patients (uveitis) for all the human work done in this report. However, the number of samples varied for each assay because we had a limited number of cells so had to prioritize the *in vitro* conditions or the measured value was outside the range of the assay. As such, the total n for each assay is designated in the figure legends. Supernatants were collected from PBMCs cultured in serum free media (SFM) without stimulation for 48-hours, uveitis patients showed significantly more IL-12 and IL-23, and significantly lower IL-10 compared to healthy donors (Fig. 3), TGF-β trended lower in uveitis patients but was not statistically significant and IL-6 and IL-1β was not significantly different (Supp Fig. 1). Some uveitis patients had more IL-6 but the overall mean was not significantly different from the healthy donors (Supp Fig. 1). While the inflammatory cytokine, TNF-α, was not statistically significant, it was undetectable in healthy controls, but was 1.9 ± 0.7 ng/mL (mean ± SD) in PBMCs from 13 uveitis patients. While IFN-γ and IL-17 were included in the analysis, IL-17 was undetectable, below the level of detection (data not shown), and IFN-γ was 2.2 ± 0.6 ng/mL in 27 uveitis patients, but was below the level of detection in controls, so not possible to make a statistical comparison (data not shown). These observations indicate the cytokines produced by PBMCs from uveitis patients are inflammatory and is skewed toward cytokine production to promote the activation and differentiation of inflammatory T cells.

**Table 1:** Age and gender of healthy controls (controls) and uveitis patients (uveitis) stratified by the type of uveitis classified as anterior, posterior, intermediate, panuveitis, or other.

	Total	Mean Age (yrs)	Age St Dev (yrs)	Gender (M)	Gender (F)
Anterior	18	47.9	16.6	7	11
Posterior	10	49.7	12.2	6	4
Intermediate	5	45.8	24.2	4	1
Panuveitis	9	51.2	13.8	3	6
Other	3	50.0	21.9	1	2
Controls	22	44.2	20.6	4	10

The other category includes neuromyelitis optica, and ocular cicatricial pemphigoid.

**Table 2:** Age and gender of healthy controls (controls) and uveitis patients (uveitis) stratified by the particular therapy.

	Total	Mean Age (yrs)	Age St Dev (yrs)	Gender (M)	Gender (F)
IMT	14	50.3	11.6	8	6
Biologics	8	44.9	14.3	6	2
Biologics + IMT	8	42.9	15.8	2	6
Steroids + IMT + Biologics + NSAIDs	11	48.7	13.4	4	7
None	4	64.3	9.4	1	3

Immunonomodulatory therapy (IMT) – methotrexate, cellcept, cyclosporine. Biologics – rituximab, adalimumab, infliximab. Non-Steroidal Anti-Inflammatory Drug (NSAID) – bromfenac. Steroids – difluprednate ophthalmic emulsion, prednisolone acetate ophthalmic suspension, loteprednol etabonate ophthalmic suspension.

### Classical monocytes are reduced and intermediate monocytes are enriched in autoimmune uveitis patients

**Figure 4:**
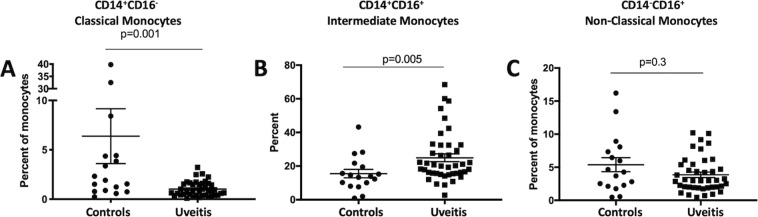
Monocyte subsets in autoimmune uveitis patients. PBMCs from uveitis patients or healthy donors were cultured for 48 hours in serum free media (SFM). Cells were stained for CD14 and CD16 and analyzed by flow cytometry. CD14 and CD16 expression was used to identify classical monocytes as CD14^+^CD16^−^ (**A**), intermediate monocytes as CD14^+^ CD16^+^ (**B**), or non-classical monocytes as CD14^−^CD16^+^ (**C**). This analysis included 44 uveitis patients (uveitis) and 17 healthy donors (controls). Significance was assessed by nonparametric Mann-Whitney U test. Statistical significance (p < 0.05) is designated by *.

In order to demonstrate further clinical relevance of our mouse findings and to focus on specific cell types we examined PBMCs from autoimmune uveitis patients. Three subsets of circulating monocytes have been identified based on expression of CD14 and CD16, classical monocytes (CD14^+^CD16^−^), intermediate (CD14^+^CD16^+^), and non-classical (CD14^−^CD16^+^) subsets^12,22,23^. Because an abnormal distribution of these monocyte subsets has been reported in other autoimmune diseases such as rheumatoid arthritis, Crohn’s disease, Eales’ disease^24,25^, we compared the distribution of these cells in uveitis patients with healthy controls. PBMCs from 42 uveitis patients and 17 healthy donors were analyzed for CD14 and CD16 expression following a 48-hour culture in SFM without stimulation. We focused on the above three subsets based on the expression of CD14 and CD16 (Supp Fig. 2). In comparison with healthy controls, uveitis patients had a significantly lower percentage of classical monocytes (Fig. 4A) but a significantly higher percentage of intermediate monocytes (Fig. 4B). In contrast, there was no significant difference in non-classical monocytes between uveitis patients and healthy controls (Fig. 4C). These observations show that the classical monocytes were lower in uveitis patients and the intermediate monocytes were higher in uveitis patients following *in vitro* culture in SFM.

### Expression of the regulatory markers, CD39 and CD73, is impaired with melanocortin-adenosinergic stimulation in monocytes from uveitis patients

We have previously published that the melanocortin-adenosinergic pathway is necessary for the induction of regulatory immunity that suppresses autoimmune uveitis in mice^12–15^. Stimulation of this pathway, specifically of the melanocortin 5 receptor (MC5r), upregulates CD39 and CD73 on macrophages^15^. CD39 and CD73 convert ATP into adenosine which stimulates the adenosine 2 A receptor on T cells to activate Tregs. Because we have previously demonstrated that monocytes and T cells from autoimmune uveitis patients express similar levels of MC5r and A2Ar compared to healthy control donors^12^, we asked if these receptors are functional. We examined if stimulation of PBMCs through the melanocortin-adenosinergic pathway upregulates CD39 and CD73 ectonucleotidase expression on monocytes.

**Figure 5:**
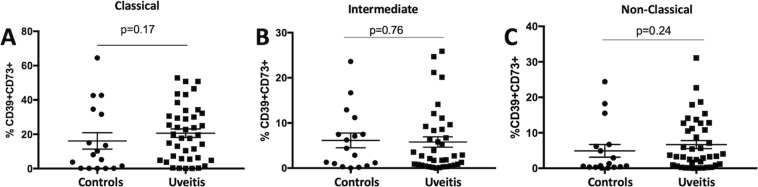
CD39 and CD73 on monocyte subsets from uveitis patients. CD39 and CD73 expression on the monocytes from Fig. 4 was determined by flow cytometry analysis. The percentage of CD39^+^CD73^+^ cells in each subset is shown from healthy controls (control, n = 15–17) and uveitis patients (uveitis, n = 37–44). Each panel is gated on the indicated monocyte subset classical (**A**), intermediate (**B**), and non-classical. (**C**) Significance was assessed by nonparametric Mann-Whitney U test.

**Figure 6:**
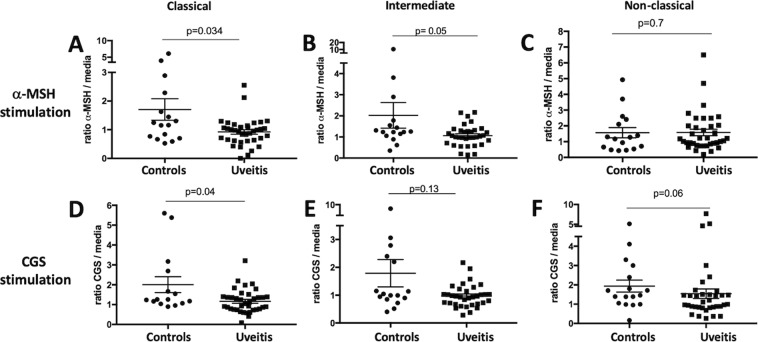
CD39 and CD73 expression on monocyte subtypes following melanocortin or A2Ar stimulation. PBMCs were collected from healthy controls (controls) or uveitis patients (uveitis) and incubated in SFM for 48 hours. Cells were incubated with 1 ng/mL α-MSH to stimulate the melanocortin receptors (**A**–**C**) or cells were incubated with 1 mM CGS21680 (CGS) to stimulate A2Ar (**D**–**F**). The percentage of CD39^+^CD73^+^ cells in each subtype was determined and because of variation from person to person, each treated sample for controls or patients were normalized against the untreated sample (media) for that same control or patient. Therefore, each panel shows the ratio of treated over untreated for controls (n = 15–17) and patients (n = 37–39) for each monocyte subtype as indicated for either α-MSH or CGS treatment. Significance was assessed by nonparametric Mann-Whitney U test. Statistical significance (p < 0.05) is designated by *.

PBMCs from patients and healthy controls were treated without (resting) or with α-melanocyte stimulating hormone (α-MSH) to stimulate melanocortin receptors (MCrs) or CGS21680 (CGS) to stimulate A2Ar for 48-hours in SFM. While some PBMCs from both controls and patients showed increases and decreases in different monocyte subsets when stimulated with α-MSH or CGS the average for the entire group remained was within 10% of the unstimulated, and there was no significant difference between the control and patient group (Supp Fig. 3). Therefore, CD39 and CD73 expression were quantified on the different monocyte subsets by flow cytometry. We focused on the monocytes that expressed both CD39 and CD73 because the two ectoenzymes work in tandem to hydrolyze ATP into adenosine. In resting PBMCs, CD39 and CD73 expression was not different between healthy controls and uveitis patients (Fig. 5A). Because there is variation in CD39 and CD73 expression from person to person, we calculated the ratio in stimulated over resting to normalize the results. When stimulated with α-MSH or CGS21680, the classical monocytes showed a significant decrease in CD39 and CD73 expression in the uveitis patients compared to the healthy controls (Fig. 6A,D). The intermediate monocytes showed a similar trend when the intermediate monocytes were stimulated with α-MSH, but it was not statistically significant (Fig. 6B). These observations show a decreased abundance of circulating classical monocytes and increased abundance of intermediate monocytes in uveitis patients compared with healthy control patients. Moreover, the classical monocytes from uveitis patients showed a significant decrease in expression of CD39 and CD73 when stimulated through MCrs or A2Ar compared with healthy controls.

### Melanocortin and A2Ar stimulation induces PD-1^+^FoxP3^+^CD25^+^CD4^+^ Tregs

We next asked if the Treg subset that was previously identified in mice that suppresses autoimmune uveitis as PD-1^+^FoxP3^+^CD25^+^CD4^+^ ^15^ (Fig. 1) can be induced through stimulation of the melanocortin-adenosinergic pathway. We therefore assayed for the abundance of PD-1^+^FoxP3^+^CD25^+^CD4^+^ Tregs cells in PBMCs of uveitis patients and healthy controls and determined if stimulation of the melanocortin-adenosinergic pathway induced Tregs.

**Figure 7:**
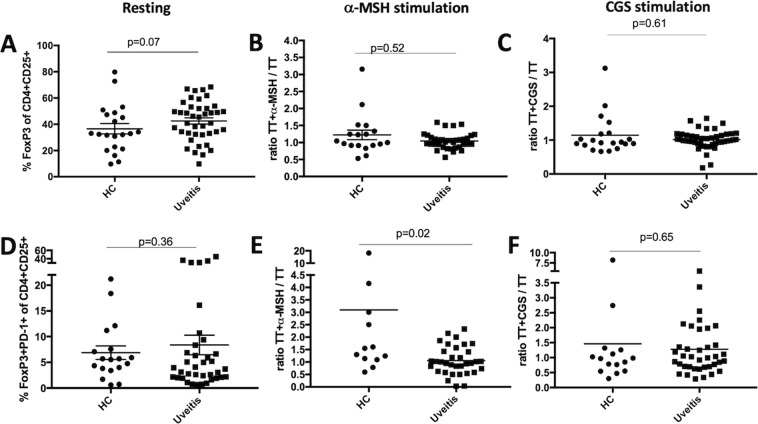
Percentage of regulatory T cells in uveitis patients. PBMCs were collected from healthy controls (HC, n = 12–18) or uveitis patients (uveitis, n = 25–34) and incubated in SFM for 48 hours. Cells were cultured in SFM alone (**A**,**D**, resting), or antigen stimulated with tetanus toxin (TT) at 1 μg/mL. Antigen stimulated cells were incubated with 1 ng/mL α-MSH to stimulate the melanocortin receptors (**B,E**) or cells were incubated with 1 mM CGS21680 (CGS) to stimulate A2Ar (**C**,**F**). Cells were analyzed by flow cytometry and the percentage of FoxP3 cells in the CD4^+^CD25^+^ population is shown (**A**–**C**) or the percentage of PD-1^+^FoxP3^+^ cells in the CD4^+^CD25^+^ population is shown (**D**–**F**). The antigen stimulated treated samples are normalized against the antigen stimulated samples (**B**,**C**,**E**,**F**). Significance was assessed by nonparametric Mann-Whitney U test. Statistical significance (p < 0.05) is designated by *.

**Figure 8:**
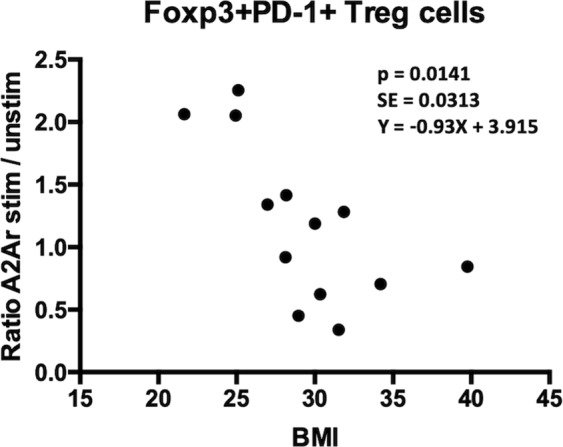
Induction of Treg cells through A2Ar stimulation in uveitis patients correlated with BMI. The ratio of FoxP3^+^PD-1^+^CD25^+^CD4^+^ A2Ar stimulated cells over unstimulated cells from uveitis patients (n = 13) that had active uveitis within the last 24 months and older than 24 years were plotted with the corresponding BMI. The age for females is 39–70 years with a mean of 46.1 years, and the age for males is 24–52 with a mean of 40.8 years. Both males and females are included because the beta estimate was similar for both. Therefore, the beta estimate is −0.093 (SE = 0.013, p = 0.0141), and is controlled for gender.

T cells were TCR stimulated with tetanus toxin (TT), which has been shown to elicit a potent T cell response^26,27^, and treated with α-MSH or CGS or cells were cultured without TCR stimulation (resting). Some patients and controls indicated if a recent tetanus vaccination had been received, but in order to screen samples that did not indicate the vaccination status or the patient was unable to recall how recent the vaccination was, we excluded samples that did not show an increase in inflammatory response in TT treated compared to resting cultures. FoxP3 expression was not significantly different between healthy controls and uveitis patients in PBMCs at rest (Fig. 7A). This FoxP3 group also only showed a moderate increase (ratio of TT stimulated over resting greater than 1.2) in the healthy controls when stimulated with α-MSH or CGS21680 (Fig. 7B,C). Therefore, because stimulation of the melanocortin-adenosinergic pathway did not elicit an appreciable FoxP3^+^ Treg response we further asked if the PD-1^+^FoxP3^+^CD25^+^CD4^+^ subset, that we observed as the functionally suppressive subset we observed in mice (Fig. 1)^15^, showed an appreciable change. The number of PD-1^+^FoxP3^+^CD25^+^CD4^+^ Tregs trended higher in the uveitis cohort under resting conditions, but was not statistically significant (Fig. 7D). While some healthy controls did not show an increase in PD-1^+^FoxP3^+^CD25^+^CD4^+^ Tregs with MC5r stimulation, the majority of controls displayed an increase of 1.2 or greater (8 out of 12), which is in stark contrast and significantly reduced in the uveitis cohort (Fig. 7E). Stimulation of A2Ar stimulation had some enhancement of the PD-1^+^FoxP3^+^CD25^+^CD4^+^ subset in healthy controls and uveitis patients but was not significantly different between the two groups (Fig. 7F). When we analyzed the body mass index (BMI) of active patients (time of collection and the last observation of uveitis is less than two years) that were over the age of 24, we found a significant correlation (Fig. 8). These observations demonstrate a significant reduction in the number of PD-1^+^FoxP3^+^CD25^+^CD4^+^ Tregs from uveitis patients when stimulated with α-MSH compared with healthy controls. Further, while there was no significant difference in the overall number of PD-1^+^FoxP3^+^CD25^+^CD4^+^ Tregs between healthy controls and uveitis patients following A2Ar stimulation with CGS21680, there were more uveitis patients that showed an increase over two-fold compared with healthy controls, and the body mass index appears to correlate with the responders. These observations show that the induction of PD-1^+^FoxP3^+^CD25^+^CD4^+^ Tregs is impaired in uveitis patients, but stimulation of A2Ar to induce this Treg population is intact. Further, response to the induction of Tregs through A2Ar stimulation may be related to BMI.

## Discussion

In this report, we sought to verify the melanocortin-adenosinergic pathway that we previously identified as important in the promotion of regulatory immunity in mice to prevent relapsing autoimmune disease is relevant in human autoimmune disease, specifically autoimmune uveitis. We show that the PD-1^+^CD25^+^CD4^+^ Treg cells require PD-1 stimulation to suppress EAU and that these Treg cells do not function to induce suppressor activity in APC through the PD-1 pathway. We next show an inflammatory cytokine profile from PBMCs cultured from uveitis patients that has significantly elevated IL-12 and IL-23 compared to controls, and significantly lower IL-10 compared to controls. Uveitis patients also had significantly more intermediate monocytes, that have been characterized as suppressive, and significantly less classical monocytes that have been characterized as inflammatory^22^. When PBMCs were stimulated through the melanocortin-adenosinergic pathway we asked if the monocytes upregulated CD39 and CD73 expression as observed in mice, and found that classical monocytes from uveitis patients had significantly lower expression of CD39 and CD73 compared to healthy controls. We further asked if there is a defect in the induction of the same Treg population we identified in mice, and found that melanocortin stimulation induced significantly fewer Tregs in the uveitis cohort compared with healthy controls. Specific stimulation of A2Ar revealed no significant change in Tregs between controls and uveitis patients and further consideration of BMI indicated a correlation with the induction of Tregs through A2Ar stimulation to BMI. These observations indicate that a block in the melanocortin pathway correlates with chronic autoimmune uveitis.

In our mouse studies we asked if the post-EAU Treg cell could induce a suppressor APC because the PD-1/PD-L1 pathway can suppress both T cells and APC^15,19,28^. However, our mouse studies indicate this is not the case, so this suggest that this pathway functions in one direction and the post-EAU Treg cell is not capable of also suppressing the APC. Based on our previous studies, the post-EAU Treg cell does inhibit the differentiation of Th1 and Th17 cells^15^, so likely functions on T cells rather than APC.

The cytokine profile expressed by PBMCs from uveitis patients showed significantly elevated IL-12 and IL-23 which are cytokines involved in polarization of the Th1 and Th17 response. An interesting finding is the decrease in IL-10 in the uveitis patients compared with controls, because IL-10 has not been observed to be involved in the generation or function of post-EAU Treg cells in mice. Instead, TGF-β is the dominant regulatory cytokine produced by post-EAU Treg cells^12–14^, which is not different between uveitis patients and controls. This finding indicates a difference between the mouse and human disease, with stimulation of the melanocortin-adenosinergic pathway resulting in a Th3 dominant Treg response in mice compared to a Tr1 dominant Treg response.

The distribution of monocyte subtypes in uveitis patients was also significantly different with less classical monocytes and more intermediate monocytes compared to controls following *in vitro* culture, so is a caveat when comparing these observations with PBMCs immediately analyzed from patients. A previous report demonstrated that glucocorticoid treatment induced intermediate (suppressor) monocytes in uveitis patients^22^, so because all the patients in this cohort are on immunosuppressive medications that include steroids, anti-metabolites, and biologics, the increase in intermediate monocytes could be due to the treatment regimen. However, it should be mentioned that there are conflicting studies demonstrating that intermediate monocytes can be non-classical monocytes or classical monocytes^29,30^. A longitudinal study is necessary to determine if this could be an indication of treatment effectiveness.

Based on our previous mouse findings that CD39 and CD73 are expressed on the post-EAU macrophage that activates regulatory T cell activity, we asked if these ectoenzymes are expressed on different monocyte subsets. Because both CD39 and CD73 are necessary to convert the proinflammatory molecule, ATP, into the regulatory molecule, adenosine^31^, we quantified the cells that expressed both. Also, in order to control for the variability from person to person we used the ratio of stimulated cells compared to unstimulated cells. Even though there was a variation in the response in controls, at least half of the controls increased expression by 20%. Furthermore, our finding that uveitis patients had a significantly lower ratio compared to controls when stimulated through either the melanocortin pathway or A2Ar in classical monocytes indicates that even though these receptors are expressed there may be a defect downstream of these receptors. The observation that there is no difference in expression of unstimulated PBMCs between uveitis patients and controls suggests that this regulatory pathway is still functional at a basal level, but the failure to upregulate upon stimulation may contribute to autoimmune uveitis.

The next step in the induction of regulatory immunity in the mouse model requires induction of Treg cells through the A2Ar stimulation by the adenosine generated by CD39^+^CD73^+^ macrophages^32,33^. Therefore, we asked if stimulation of this pathway can induce Treg cells in PBMCs from uveitis patients. There was no significant difference in total FoxP3^+^ Tregs, at rest or when stimulated through melanocortin receptors or A2Ar. However, when we quantified the number of PD-1^+^FoxP3^+^ Tregs as we found to be the suppressive Treg population in mice, we found a significant decrease in PBMCs from uveitis patients compared to controls when stimulated through the melanocortin pathway, but not when stimulated through A2Ar. This suggests that in contrast to our mouse findings, melanocortin stimulation in humans is sufficient to induce Tregs. A2Ar stimulation did increase the number of Tregs by 25% in controls, suggesting a variability with response to A2Ar that is also present in uveitis patients. We further asked if the uveitis patients that did respond to A2Ar stimulation could be linked with body mass index, and found a significant correlation. This last observation suggests a potential link with autoimmune uveitis and obesity and is not unexpected given multiple reports linking obesity with autoimmune diseases^34–37^.

The prevalence of uveitis is slightly greater in females, which was observed in our study with the exception of intermediate uveitis. However, a limitation of this study is the low sample size for this type of uveitis. When we stratified the patients based on the type of treatment we observed the opposite gender ratio in patients on IMT or Biologics alone, with more female patients receiving multiple therapies. Because patients requiring multiple classes of medication relates to the severity of disease or recalcitrant disease, this reinforces previous observations of a higher female prevalence. Although we did not observe any differences between treatment groups we did not stratify the type of uveitis with the type of treatment, but these observations support the need for a larger study in the future to address if the type of uveitis and type of treatment has an impact on the induction of Tregs through the melanocortin-adenosinergic pathway.

This study demonstrates that the post-EAU PD-1^+^FoxP3^+^CD25^+^CD4^+^ Tregs that are generated through the melanocortin-adenosinergic pathway, functionally suppress EAU and that they do not convert APC to become suppressive. We further show that there may be a defect in the ability of monocytes to respond to melanocortin stimulation, which may contribute to disease. Because it has been demonstrated that Tregs are induced through the melanocortin pathway^38,39^, this could be due to a decreased ability to induce Tregs. However, bypassing melanocortin stimulation to stimulate A2Ar directly, generated Treg cells, and the patients that responded to A2Ar had lower BMI. It has been recently demonstrated that EAU is exacerbated by a high fat diet in mice^40^. However, while this is suggestive that patients with lower BMI are more likely to response to A2Ar stimulation, additional mechanistic studies are necessary to better demonstrate a connection. We and others have demonstrated that Treg cells are necessary for resolution of EAU and for prevention of relapse^12,14–16,40,41^. As such, this study complements previous animal studies and suggests that autoimmune uveitis may be due to an inability of classical monocytes to respond to melanocortin stimulation.

## Methods

### Mice

All mouse procedures described in this study were approved by the University of Oklahoma Health Sciences Center Institutional Animal Care and Use Committee (OUHSC IACUC) and all mouse study methods were carried out in accordance with the relevant guidelines approved by the OUHSC IACUC. C57BL/6 J mice were purchased from Jackson Laboratories.

### Experimental autoimmune uveoretinitis (EAU)

Mice between 6–8 weeks old were immunized for EAU as previously described^15^. We used male mice because in our facility, we have not observed significant differences in this disease model between male and female mice. Briefly, an emulsion of complete Freund’s adjuvant (CFA) with 5 mg/mL desiccated *M. tuberculosis* (Difco Laboratories, Detroit, MI) and 2 mg/ml interphotoreceptor retinoid binding protein (residues 1–20) (IRBP) (Genscript, Piscataway, NJ) was used to immunize mice for EAU. A volume of 100 μL of the emulsion was injected subcutaneously at two sites in the lower back followed by an intraperitoneal injection of 0.3 µg pertussis toxin. The severity of retinal inflammation during the course of EAU was evaluated every 3–4 days by fundus examination using a slit lamp microscope. Before examining the retina, the iris was dilated with 1% tropicamide, the cornea was numbed with 0.5% proparacaine, and the cornea was flattened with a glass coverslip in order to examine the retina. The clinical signs of observable infiltration and vasculitis in the retina were scored on a 5-point scale as previously described^42^. Both eyes were scored and the higher score was used to represent that mouse for that day, the average score for the group of mice was then calculated.

### *In Vitro* stimulation

Post-EAU spleens (day 90–100 after immunization for EAU) were collected into 5% FBS in RPMI supplemented with 10 μg/ml Gentamycin (Sigma), 10 mM HEPES, 1 mM Sodium Pyruvate (BioWhittaker), Nonessential Amino Acids 0.2% (BioWhittaker) and made into a single cell suspension that was depleted of red blood cells using RBC lysis buffer (Sigma, St Louis, MO). The spleen cells were resuspended in serum free media (SFM) and IRBP was added at 50 μg/mL for 48 hours at 37 °C and 5% CO_2_ to reactivate antigen specific T cells. SFM consisted of RPMI-1640 with 1% ITS + 1 solution (Sigma) and 0.1% BSA (Sigma). PD-1 neutralizing antibody (clone J43, ebioscience) was added during reactivation to block the PD-1/PD-L1 pathway where indicated. Following reactivation, cells were collected for adoptive transfer of 1 × 10^6^ cells by tail vein injection into recipient mice at the same time as immunization for EAU.

### Cell sorting for post-EAU treg cells

Reactivated spleen T cells from post-EAU mice were stained as described above. Antibodies used to stain T cells were anti-CD4 (clone GK1.5, Biolegend, San Diego, CA), anti-CD25 (clone PC61, Biolegend), and anti-PD-1 (clone 29 F.1A12, Biolegend). Gating was determined using single stained samples. Stained cells were sorted in the Oklahoma Medical Research Foundation Flow Cytometry Core on a FACSAria III (BD Biosciences). Cells were sorted into tubes containing 10% FBS and found to be ≥98% pure.

### APC co-culture

Adherent spleen APC were obtained by incubating an RBC depleted single cell splenocytes on polystyrene for 90 minutes at 37 °C and 5% CO_2_ in 10% FBS/RPMI. Non-adherent cells were removed and adherent cells were scraped off with ice cold 10% FBS/RPMI. Adherent cells were then washed with SFM and incubated with sorted Treg cells for 48 hours at a 1:1 ratio. Following the 48-hour co-culture cells were collected and CD11b^+^ APC were sorted by magnetic positive selection (StemCell Technologies, Vancouver, Canada). Popliteal lymph nodes were collected from mice that received a foot pad injection of CFA/IRBP seven days earlier. CD4^+^ T cells were enriched using a CD4 T cell enrichment kit (R&D Systems) and added to the sorted CD11b^+^ APC at 2:1 (T cell:APC) for 48-hours. After 48-hours the entire cell mixture was collected and 1 × 10^6^ cells were transferred to recipient EAU mice as described above.

### Human studies

Institutional Review Board Approval was obtained from New England IRB, and the University of Oklahoma Human Research Participant Protection IRB to collect and analyze PBMC from human uveitis patients. All experiments involving human subjects were carried out in accordance with the relevant guidelines approved by the appropriate IRB. Prior to the collection of patient samples informed consent was obtained from all subjects. Patient samples were de-identified and limited patient information was made available to the research staff.

### Peripheral blood mononuclear cell (PBMC) preparation

Whole blood was collected from patients by the clinical staff at MERSI (Waltham, Massachusetts) or DMEI (Oklahoma City, OK), and some healthy control samples were obtained from Oklahoma Blood Institute (Oklahoma City, OK). Within 24 hours from the time of collection the blood was processed using SepMate 50 tubes (Stemcell Technologies, Vancouver, BC, Canada) to isolate the PBMCs from the blood. PBMCs were then immediately cultured *in vitro* at 1 × 10^6^ cells per well in a 24-well plate as described below.

### PBMC cultures

The A2Ar agonist, CGS21680 (CGS) (Tocris, Bristol, U.K.) in DMSO (27.7 mg/mL) was diluted in PBS to a final concentration of 0.05 mg/mL. For monocyte assays, α-MSH (Bachem, Vista, CA) at 1 ng/mL and/or CGS (1 μM/well) were added into separate wells. T cells were antigen stimulated with Tetanus Toxoid (TT) (List Biologicals Laboratories, Campbell, CA) at 1 μg/mL was added to cultures. PBMC cultures were maintained in humidified incubator at 37 °C in 5% CO_2_ for 48 hrs in SFM. At the end of the incubation time, PBMCs were collected by centrifugation (300 g for 5 mins). PBMCs were analyzed with flow cytometry and supernatants were saved for cytokine assays.

### Cytokine analysis

Cell culture supernatants were assayed using the human Th1/Th2/Th17/Th22/Treg 18-multiplex procartaplex kit, (Invitrogen, Vienna, Austria) was used to assay the PBMC culture supernatants. The Multiplex plate was analyzed with Bio-Rad plate reader (Bioplex system,Hercules, CA). The assay was performed according to manufacturer’s instructions. The TGF-β concentration was measured with the standard Mv1Lu bioassay^43^.

### Flow cytometry

Human PBMCs were stained with anti-CD14 (clone M5E2, Biolegend), anti-CD16 (clone 3G8, Biolegend), anti-CD4 (clone OKT4, Biolegend), human CD39 (clone 498403, R&D Systems), human CD73 (clone 606112, R&D Systems)^44,45^, human CD25 (clone BC96, Biolegend), human FoxP3 (clone 236 A/E7, Biolegend), and human PD-1 (clone EH12.2H7, Biolegend). Prior to anti-FoxP3 staining, the cells were fixed and permeabilized.

Stained cells were analyzed in the Oklahoma Medical Research Facility (OMRF) Flow Cytometry Core Facility on a BD LSRII (BD Biosciences) and data was analyzed using FlowJo Software (Tree Star, Inc., Ashland, OR). Single stained samples were used to determine compensation values, and fluorescence minus one (FMO) controls were used to determine gating placement.

### Statistics

Statistical significance between EAU scores was determined using nonparametric Mann-Whitney U test between groups of mice. Two-way ANOVA was also used to assess significant changes in the tempo of disease between the groups of treated EAU mice. Cytokine concentrations were statistically analyzed by one-way ANOVA with post-test Bonferroni comparison analysis. Statistical significance was determined when P ≤ 0.05 (two-sided). Healthy controls (*n* = 22) were compared against patients with uveitis (*n* = 45) across a range of selected biomarkers. Each comparison was conducted via a Mann-Whitney U test using a per-test alpha level of 0.05. Nonparametric methods were used because the data were largely right-skewed for each biomarker. All statistical tests used to analyze human samples were conducted in R v3.5.1 and statistical analysis for mouse experiments were analyzed with Graphpad Prism software.

## Supplementary information

Supplementary Figures
