# Supplementary material for: PD-1+ melanocortin receptor dependent-Treg cells prevent autoimmune disease
Source: Sci Rep. 2019 Nov 15;9:16941. doi: 10.1038/s41598-019-53297-w (PMC6858311; doi:10.1038/s41598-019-53297-w)
Supplement: Supplementary file 1 — Supplementary Figures [file 41598_2019_53297_MOESM1_ESM.pdf]

PD-1^{+} melanocortin receptor dependent-Treg cells prevent autoimmune disease

Fauziyya Muhammad[1], Dawei Wang[2], Alyssa Montieth[3,4], Stacey Lee[3,4], Janine Preble[3,4], C. Stephen Foster[3,4,5], Theresa A. Larson[2], Kai Ding[6], Justin D. Dvorak[6], and Darren J. Lee[1,2*]

Affiliations:

[1]Department of Microbiology and Immunology, University of Oklahoma Health Sciences Center, Oklahoma City, Oklahoma, USA

[2]Department of Ophthalmology/Dean McGee Eye Institute, University of Oklahoma Health Sciences Center, Oklahoma City, Oklahoma, USA

[3]Massachusetts Eye Research and Surgery Institute, Waltham, Massachusetts, USA.

[4]Ocular Immunology and Uveitis Foundation, Waltham, Massachusetts, USA.

[5]Harvard Medical School, Boston, Massachusetts, USA

[6]College of Public Health, University of Oklahoma Health Sciences Center, Oklahoma City, OK, USA

*Corresponding author

Darren J. Lee, PhD

Supplementary Materials

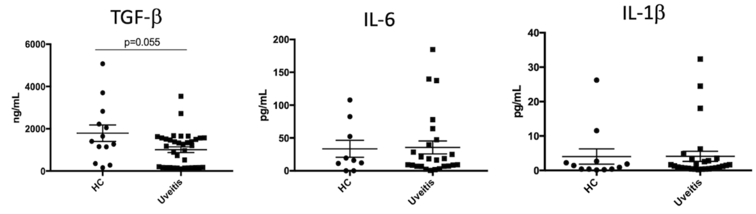

Supp Figure 1

Supplementary Figure 1. Cytokine profile in PBMCs from uveitis patients. PBMCs from uveitis patients or healthy donors were cultured for 48 hours in serum free media. After the 48-hour culture the supernatants were collected and cytokines were measured by Bioplex. The cytokines that were not significantly different between PBMCs from health controls (HC) and uveitis patients (uveitis) is shown. The regulatory cytokine TGF-β is shown HC (n = 13) and uveitis (n = 36), the proinflammatory cytokines IL-6 HC (n = 9) and uveitis (n = 25), and IL-1β HC (n = 12) and uveitis (n = 28) are shown. Significance was assessed by nonparametric Mann-Whitney U test.

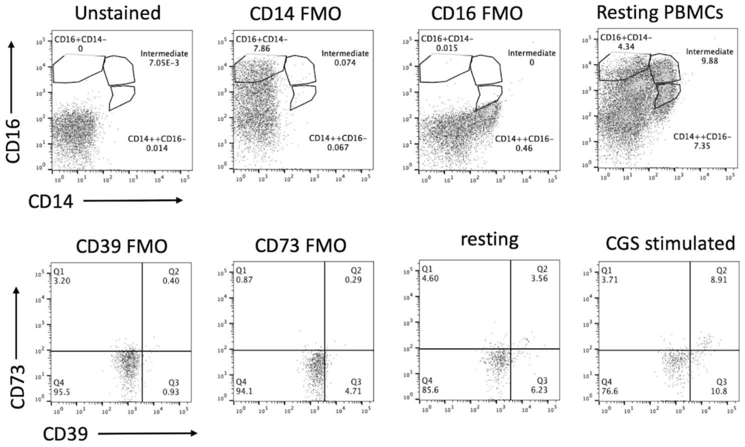

Supp Figure 2

Supplementary Figure 2. Representative flow cytometry graph of PBMCs stained for CD14 and CD16. PBMCs from healthy donors were cultured for 48 hours in serum free media (SFM). Cells were stained for CD14 and CD16 and analyzed by flow cytometry. CD14 and CD16 expression was used to identify classical monocytes as $CD14^{+}CD16^{-}$, intermediate monocytes as $CD14^{+}$ $CD16^{+}$, or non-classical monocytes as $CD14^{-}CD16^{+}$.

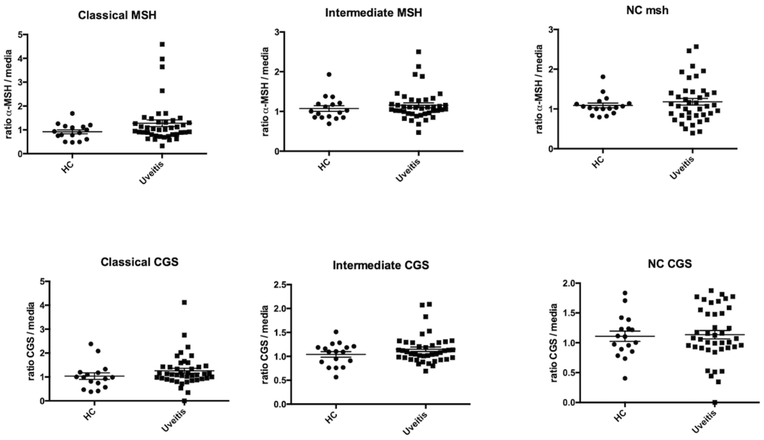

Supp Figure 3

Supplementary Figure 3. Monocyte distribution following melanocortin or A2Ar stimulation. PBMCs were collected from healthy controls (controls) or uveitis patients (uveitis) and incubated in SFM for 48 hours. Cells were incubated with 1 ng/mL α-MSH (MSH) to stimulate the melanocortin receptors or cells were incubated with 1 mM CGS21680 (CGS) to stimulate A2Ar. The percentage of monocytic subtype, classical, intermediate, or non-classical (NC) was determined and because of variation from person to person, each treated sample for controls or patients were normalized against the untreated sample (media) for that same control or patient. Therefore, each panel shows the ratio of treated over untreated for controls (n = 16-17) and patients (n = 40-42) for each monocyte subtype as indicated for either α-MSH or CGS treatment. Significance was assessed by nonparametric Mann-Whitney U test.
